# Acceptance of a Cognitive Stimulation Web lab within MCI Patients

**DOI:** 10.1002/alz70858_105066

**Published:** 2025-12-26

**Authors:** Leticia Vivas, Claudia Arias, María de la Paz Pereyra, Elsa Araceli Revollo Sarmiento, Maria Celeste López Moreno, Axel Fernández‐Zaionz

**Affiliations:** ^1^ Institute of Basic and Applied Psychology and Technology (IPSIBAT), National University of Mar del Plata (UNMDP), Psychology Faculty, Mar del Plata, Buenos Aires, Argentina; ^2^ National Council of Scientific and Technical Research (CONICET), Mar del Plata, Buenos Aires, Argentina; ^3^ Faculty of Health Sciences and Social Work of the National University of Mar del Plata (UNMDP), Mar del Plata, Buenos Aires, Argentina

## Abstract

**Background:**

The cognitive stimulation web lab LABPSI has been recently developed and designed following the User Experience framework to be used by older adults with and without cognitive impairment. The project aims to analyze its acceptance for individual use by MCI subjects in two settings: in person and at home.

**Method:**

Eight MCI participants were included in this study at this stage. Six of them performed the activities in person at their usual cognitive stimulation workshops and two performed the activities at home. Both groups were instructed to do exercises in the LABPSI alone, but they could ask for help navigating the site if needed. Participants were assessed at the start with the Fototest and a Digital Competencies Questionnaire. Then they were asked to perform certain exercises of LABPSI during eight sessions of 45 minutes twice a week. After that they were assessed again with the Fototest and an acceptance questionnaire. The questionnaire included a 5‐point Likert scale with questions about 4 dimensions: ease of use, interface and satisfaction, perceived usefulness and contents. The interview ended with open questions on the same dimensions.

**Result:**

Based on a full scale of 1 to 5, participants' scores ranged between 3 (neither agree nor disagree) and 5 (totally agree) with mean scores of: ease of use: 4.30, interface and satisfaction: 4.49, perceived usefulness: 4.53, and contents: 4.84. Responses to open questions were very positive with a good perception of the LABPSI in general, highlighting LABPSI's usefulness, ease of use, and diverse content. They also said they would recommend it to others. There were no differences between in person and at home settings, although the examiners observed it was harder to maintain participation with at home users.

**Conclusion:**

The LABPSI showed to be a well accepted technological tool to perform cognitive stimulation activities both in person and at home. Participants gave positive comments and scores on the acceptability scale were mainly high. As a qualitative data it was observed that some additional help is needed for MCI participants to perform the activities at home on their own.

